# Influence of different irrigation methods on the alfalfa rhizosphere soil fungal communities in an arid region

**DOI:** 10.1371/journal.pone.0268175

**Published:** 2022-06-17

**Authors:** Qizhang Deng, Yong Wu, Xiang Zhao, Chengshu Qiu, Shan Xia, Yuanyuan Feng, Hongling Liu

**Affiliations:** 1 College of Life Sciences, Shihezi University, Shihezi, Xinjiang, China; 2 College of Chemistry and Life Sciences Sichuan Provincial Key Laboratory for Development and Utilization of Characteristic Horticultural Biological Resources, Chengdu Normal University, Chengdu, China; ICAR-National Rice Research Institute, INDIA

## Abstract

Xinjiang is the largest arid and saline agricultural region in China. The common irrigation methods in this area are traditional flood irrigation and drip irrigation. In this study, we investigated the effects of these two irrigation methods on the fungal diversity, community structures, and functions in alfalfa rhizosphere soil as well as the associated environmental factors in northern Tianshan Mountain (Xinjiang, China). Soil enzyme activities (urease and neutral phosphatase) were significantly higher in the drip-irrigated alfalfa rhizosphere soil than in the flood-irrigated alfalfa rhizosphere soil, whereas the fungal alpha diversity in the drip-irrigated alfalfa rhizosphere soil was significantly lower than that in the flood-irrigated alfalfa rhizosphere soil. Six dominant fungal phyla were identified (>0.1%), with Ascomycota being the most abundant in all soils, followed by Basidiomycota (5.47%), Mortierellomycota (1.07%), Glomeromycota (0.55%), Rozellomycota (0.27%), and Chytridiomycota (0.14%). Ascomycota and Glomeromycota species were significantly less abundant in drip-irrigated alfalfa rhizosphere soil than in flood-irrigated alfalfa rhizosphere soil. A LEFSe analysis identified Cladosporiaceae (20.8%) species as the most abundant marker fungi in drip-irrigated alfalfa rhizosphere soil. Of the 13 fungal functional groups identified on the basis of the functional annotation using the FUNGuild database, Ectomycorrhizal (22.29%) was the primary functional group. Compared with flood irrigation, drip irrigation significantly decreased the relative abundance of Ectomycorrhizal and Arbuscular_Mycorrhizal, while increasing the relative abundance of Plant_Pathogen, although not significantly (P = 0.19). Available potassium was revealed to be the main environmental factor influencing soil enzyme activities, fungal alpha diversity, fungal community structures, and fungal functions in response to the different irrigation methods. In conclusion, drip irrigation may be more appropriate than flood irrigation in the Tianshan dryland agricultural area for enhancing soil enzyme activities, but it may also increase the abundance of plant pathogenic fungi in the soil.

## Introduction

Alfalfa (*Medicago sativa* L.), which is a perennial leguminous forage crop with a long history of cultivation, is high yielding, nutritious, and palatable for livestock, but it is also tolerant to drought and salinity and is highly adaptable, making it suitable for the northern and western arid and semi-arid regions of China [1, 2]. However, a serious lack of water resources, high water consumption, and the low water use rate of traditional flood irrigation in these areas have seriously limited alfalfa yield and quality. Therefore, decreasing water waste to maximize the use of the limited water resources and improving the water use efficiency of plants are crucial for enhancing the large-scale production of crops in arid areas.

Drip irrigation is a highly efficient water-saving irrigation technology that can overcome the complex topographical constraints of mountainous and hilly areas to deliver water and nutrients quantitatively and uniformly to the soil around crop roots. Additionally, it has been widely used in dryland agroecosystems as an alternative to traditional flood irrigation to improve productivity in terms of water use efficiency and yield increases [3, 4]. Related studies revealed that compared with flooding, drip irrigation decreases surface evaporation losses, increases crop water productivity, and significantly improves plant physiological and growth indicators [5–7]. In addition to affecting plant growth and development, drip irrigation also modifies soil nutrient contents and soil microbial community structures [8]. Drip irrigation strips are buried in soil. Thus, their depth under certain soil matrix conditions affects the transport and distribution of soil water, which in turn affects the soil microstructure, soil nutrient transport and distribution, soil microorganism growth and distribution [9], plant root growth, and crop nutrient uptake [10]. Soil microbial communities are important for the biochemical cycling of materials and energy flow, which is critical for soil ecosystems. Moreover, soil microorganisms are very sensitive to environmental changes in soil ecosystems [11]. Because changes in community structures can reflect the stability of the soil quality, crop yield, and soil ecosystems to some extent, they are often used as sensitive indicators of soil health [12]. Among soils, plant rhizosphere soil interacts the most with plants. Hence, it is a critical site for root, soil, and microbial interactions, energy flow, and material cycling, making it important for the conversion, transport, and uptake of soil nutrients related to plant growth [13]. Different irrigation practices can differentially affect soil moisture and alter microbial community functions and structures [14, 15], but little is known about the effects of various irrigation practices on the diversity, community structures, and functions of microbes and the activities of enzymes in the rhizosphere soil of crops, especially in the Tianshan agricultural region in China.

Located in the Eurasian hinterland, Xinjiang Tianshan is a large mountain range that is surrounded by a huge desert. Its many natural features include mountain–basin landscape patterns, numerous glaciers and rivers, and special biota and ecological processes; it is the most typical representative of large mountain ecosystems in the global temperate arid zone [16]. Because alfalfa is an economically important agricultural crop in Xinjiang, clarifying the effects of drip irrigation on the alfalfa rhizosphere microorganisms and their interactions with soil environmental factors may be useful for developing methods enabling the production of high-quality alfalfa and the sustainable use of soil and water resources in arid areas. Fungi are important soil microorganisms that are often used as indicators of soil fertility. Many soil fungi are plant pathogens that often cause severe damages. There are also numerous mycorrhizal fungi that have symbiotic relationships with plants (e.g., arbuscular mycorrhizal fungi), with important roles related to soil nutrient activation and plant growth and development. In this study, we analyzed the structural diversity of the alfalfa rhizosphere fungal community in response to drip irrigation and traditional flood irrigation. Additionally, we determined the physicochemical properties of alfalfa rhizosphere soil and explored how soil fungal communities and enzyme activities are influenced by soil environmental factors in the Shihezi agricultural zone of Tianshan Mountain in northern Xinjiang, China.

The objectives of this study were as follows: (1) to reveal the effects of drip irrigation on soil enzyme activities in the alfalfa rhizosphere; (2) to clarify the effects of drip irrigation on the diversity, community structures, and functions of soil fungi in the alfalfa rhizosphere; and (3) to explore the soil environmental conditions affected by different irrigation methods that influence the soil fungal community and soil enzyme activities in the alfalfa rhizosphere.

## Materials & methods

### Sample collection and processing

The test site (Company 11 of the 147th Xinjiang Shihezi Regiment) was located in the middle section of the northern foot of Tianshan Mountain, Xinjiang (85°25′57″–86°8′12″E, 44°19′34″–44°24′52″N), with an altitude between 358.6 and 360.7 m above sea level. The area has a typical continental arid and semi-arid climate, with an annual rainfall of 125–208 mm and an average annual temperature of 6.9°C. The main crops grown in the region are alfalfa, wheat, and cotton.

Alfalfa plants were grown for 3 years using drip irrigation (at a depth of 10 cm) and traditional flood irrigation methods. Three standard sample plots (20 × 20 m) were set up for each irrigation method. Aside from the irrigation, all conditions were the same. During the whole alfalfa fertility period, the total irrigation volume for drip irrigation was 6,750 m^3^/hm^2^, whereas the total irrigation volume for flood irrigation was 9,000 m^3^/hm^2^. The irrigation volume was determined on the basis of the local irrigation practices and the results of previous research to ensure sufficient water was provided to the growing alfalfa plants. Samples were collected 6 days after irrigation, using the S-shaped five-point mixed sampling method. Soil tightly bound to the alfalfa root system (within 0–4 mm of the roots) was collected using the root shaking method and designated as rhizosphere soil. Soil at the same depth as the rhizosphere soil, but without any nearby plant roots was used as the non-rhizosphere soil [17]. A total of 12 samples were collected from the drip-irrigated alfalfa rhizosphere soil (DIR), the drip-irrigated alfalfa non-rhizosphere soil (DIB), the flood-irrigated alfalfa rhizosphere soil (CKR), and the flood-irrigated alfalfa non-rhizosphere soil (CKB). The collected soil samples were stored at 4°C and immediately transported to the laboratory to be divided into three parts: one was stored at −80°C for high-throughput sequencing, one was stored at 4°C for a soil enzyme activity analysis, and one was air-dried and stored for an analysis of physicochemical properties.

### Soil physicochemical properties and enzyme activity analyses

The following soil physicochemical properties were determined as previously described [18]: soil organic matter (SOM) content (determined according to an external heating method), electrical conductivity (EC) and pH (soil:water ratio 1:2.5, determined using the OHAUS test pen), soil water (SW) content (determined according to the aluminum box weighing method), total potassium (TK) content (determined by flame photometry), total phosphorus (TP) content (determined according to the HClO_4_–H_2_SO_4_ method), total nitrogen (TN) content (determined according to the semi-micro Kjeldahl method), available potassium (AK) content (determined according to the NH_4_OAc leaching–flame photometry method), available nitrogen (AN) content (determined according to the alkaline diffusion method), and available phosphorus (AP) content (determined according to the 0.5 mol/L NaHCO_3_ method).

The activities of the following enzymes were determined: soil neutral phosphatase (SNP) [determined using a commercial kit (Solarbio® Technology Co., Ltd., China)] and soil urease (SUE) (determined by sodium phenol–sodium hypochlorite colorimetry) [19].

### Soil DNA extraction, PCR amplification, and high-throughput sequencing

Genomic DNA was extracted from samples according to the CTAB (Nobleryder, China) method. The concentration and purity of the DNA were determined by agarose gel electrophoresis. Appropriate DNA samples were transferred to a centrifuge tube and diluted to 1 ng/μL by adding sterile water. Three replicates were prepared per sample. The diluted genomic DNA was used as a template for a PCR amplification. The primers for fungi were ITS5-1737F (5′-GGAAGTAAAAGTCGTAACAAGG-3′) and ITS2-2043R (5′-GCTGCGTTCTTCATCGATGC-3′), which were specific for the ITS1 region. The PCR solution comprised 15 μL 2× Phusion Master Mix, 3 μL (each) 2 μmol/L primer, 7 μL 1 ng/μL genomic DNA, and 2 μL ddH_2_O. The PCR program was as follows: 98°C for 1 min; 30 cycles of 98°C for 10 s, 50°C for 30 s, and 72°C for 30 s; 72°C for 5 min. The PCR products were analyzed by 2% agarose gel electrophoresis, and the target bands were recovered using the GeneJET Gel Recovery Kit (Thermo Scientific, USA) and then purified using the Ion Plus Fragment Library Kit (Thermo Fisher, USA). The sequencing library was constructed using the Ion Plus Fragment Library Kit, quantified by Qubit, and tested for library compliance before being sequenced on the Ion S5™ XL system (Thermo Fisher).

### Data processing and analysis

The Ion S5™ XL sequencing data were in the fastq format. The data for each sample were split by barcodes using the Cutadapt software [20] to remove primer sequences, barcodes, and chimeric sequences [21]. Regarding the retained high-quality valid data, the sequences with 97% identity were clustered into operational taxonomic units (OTUs) using the Uparse program [22]. The Unite database was used for species annotations of the representative sequences of individual OTUs. Differences in the fungal community composition among the soil samples were determined according to the relative abundance of soil fungal communities at the phylum level. A linear discriminant analysis effect size (LEfSe) analysis at the family level was performed to identify significantly different biomarkers. The ANOVAs were performed using the agricolae package of the R program, with *post hoc* tests conducted according to the LSD method. A Pearson correlation analysis, Mantel test, and principal coordinates analysis (PCoA) were completed using the linkNT package and the vegan package in R. Canoco 5 was used for the redundancy analysis performed to investigate the relationship between soil fungal community structures and soil physicochemical factors. A 999-rank Monte Carlo permutation test was used to determine the significance of the soil physicochemical factors.

## Results

### Soil enzyme activities and physicochemical properties

**Fig 1:**
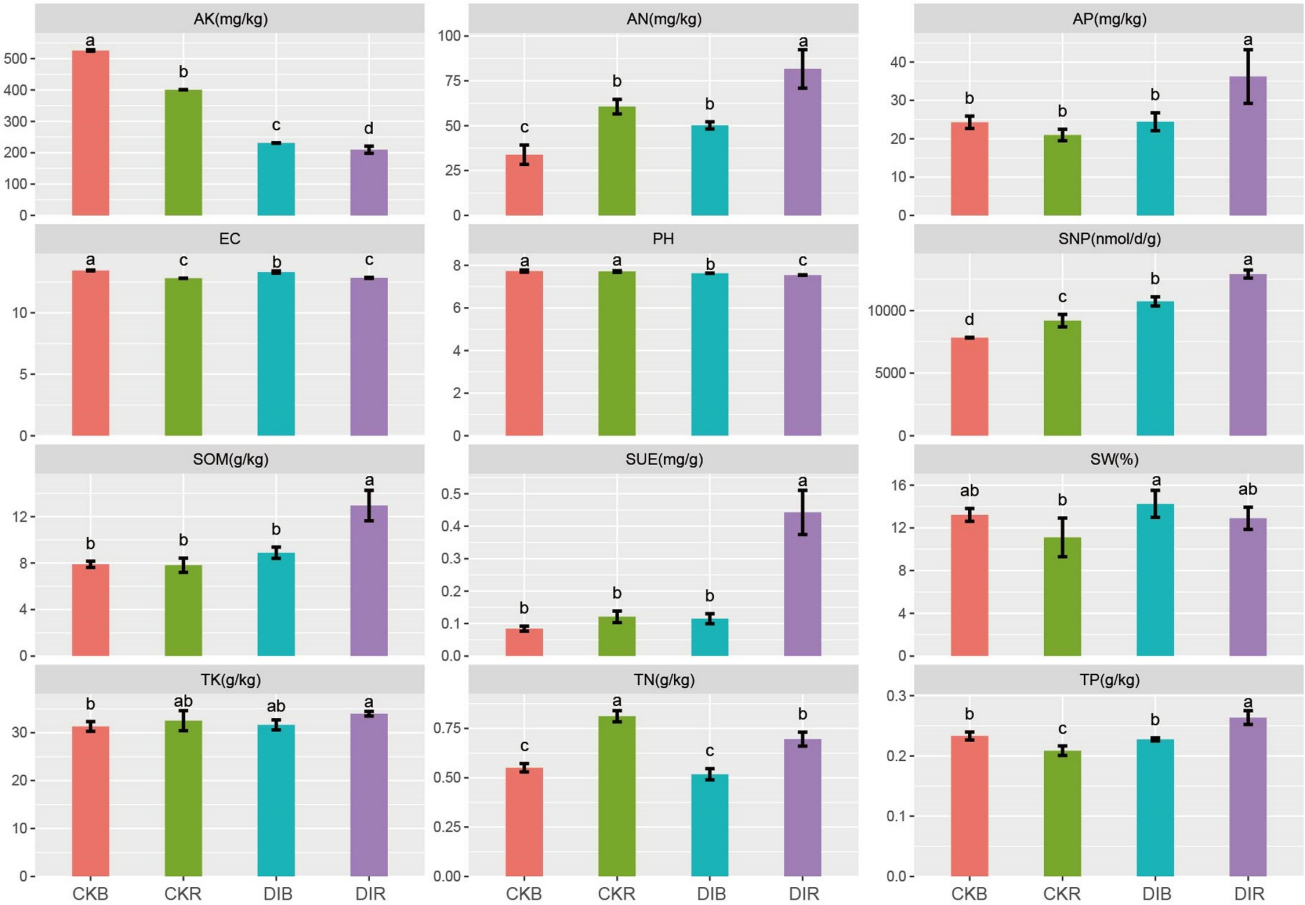
Soil physicochemical properties and enzyme activities. DIR: drip-irrigated alfalfa rhizosphere soil; DIB: drip-irrigated alfalfa non-rhizosphere soil; CKR: flood-irrigated alfalfa rhizosphere soil; CKB: flood-irrigated alfalfa non-rhizosphere soil; AK: available potassium; AN: available nitrogen; TK: total potassium; EC: electrical conductivity; SOM: soil organic matter; TN: total nitrogen; AP: available phosphorus; TP: total phosphorus; SW: soil water; SUE: soil urease; SNP: soil neutral phosphatase. Different lowercase letters indicate significant differences (P < 0.05).

The enzyme activities and physicochemical properties of DIR, DIB, CKR, and CKB are provided in Fig 1. The SOM content, SUE activity, SNP activity, SW content, AN content, AP content, TP content, and TK content were significantly higher in DIR than in CKR. The pH, AK content, and TN content were significantly lower in DIR than in CKR.

Regarding the drip-irrigation effects, the TN, TP, TK, AN, and SOM contents and the SUE and SNP activities were significantly higher in DIR than in DIB, but the pH, AK content, and EC were significantly lower in DIR than in DIB. The SUE and SNP activities can be used to evaluate the extent of the N and P biotransformations in soil. The data indicated that the nutrient contents (except AK and TN) and soil enzyme activities were significantly higher in drip-irrigated alfalfa soil than in flood-irrigated alfalfa soil. Additionally, the soil pH was lower for the drip-irrigated soil than for the flood-irrigated soil.

### Effect of different irrigation methods on the diversity of fungal communities

**Fig 2:**
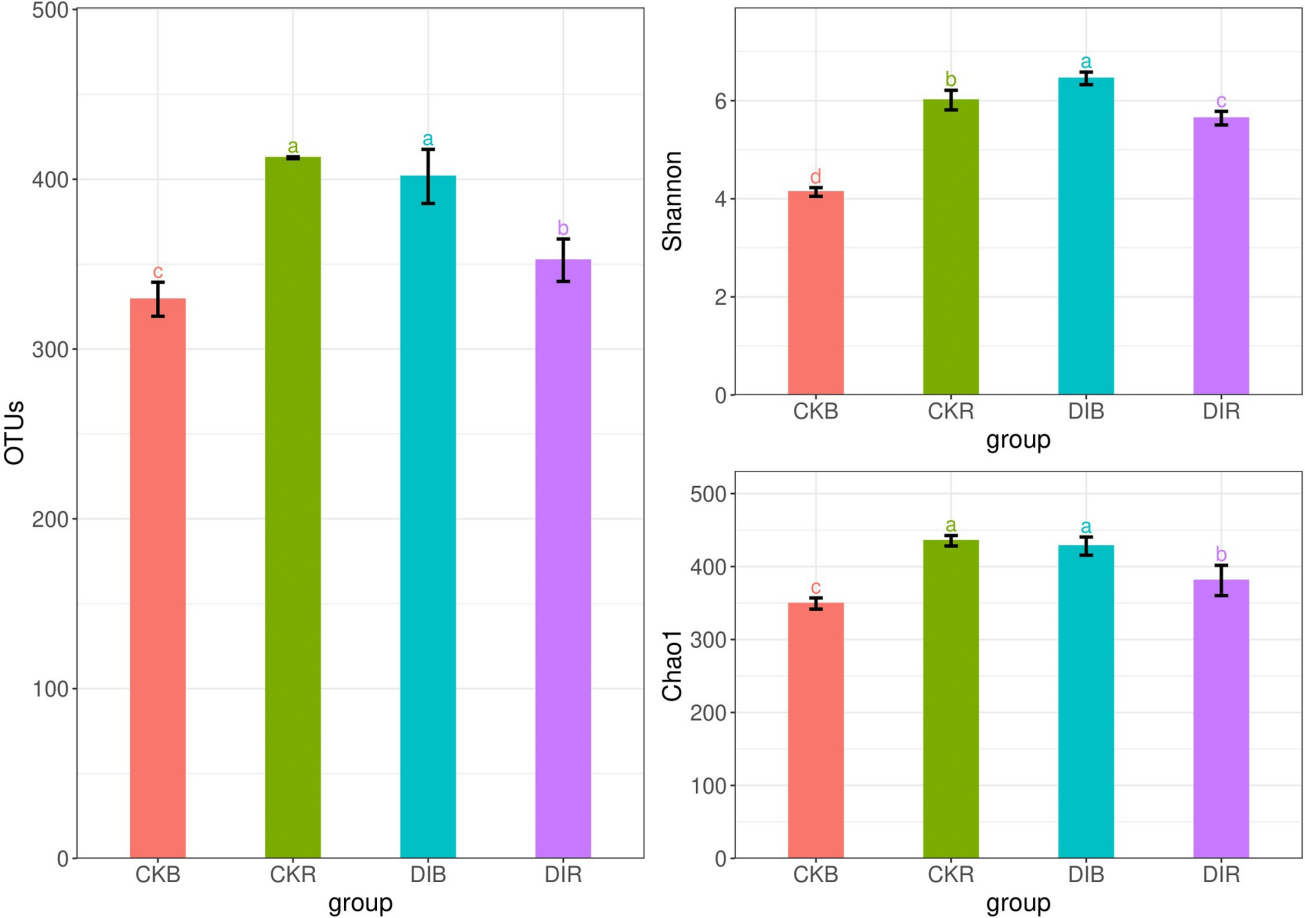
Abundance and diversity of fungal communities in differentially irrigated alfalfa soils. Different lowercase letters indicate significant differences (P < 0.05).

**Fig 3:**
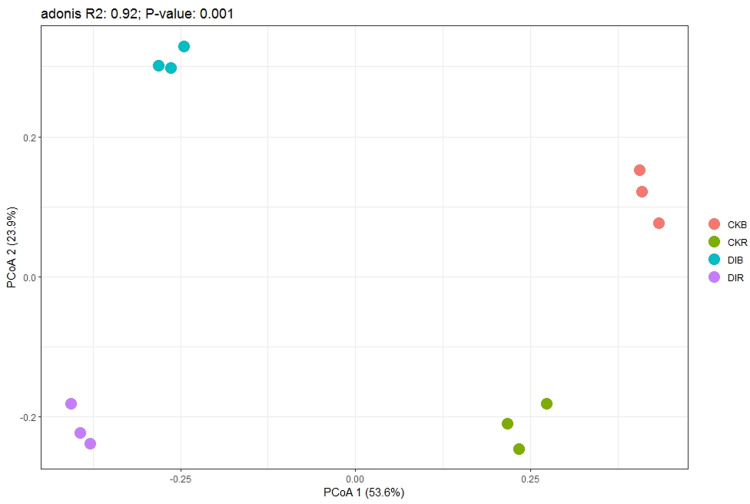
PCoA of soil fungal communities in drip-irrigated and flood-irrigated soils used to grow alfalfa (beta diversity).

There was a significant difference in the fungal diversity index between drip and flood irrigation (Fig 2). In the rhizosphere soil, the OTUs, Shannon index, and Chao1 index were significantly lower for drip irrigation than for flood irrigation. Hence, compared with the flood irrigation effects, drip irrigation significantly decreased the soil fungal diversity. In response to drip irrigation, the OTUs, Shannon index, and Chao1 index were significantly lower in the rhizosphere soil than in the non-rhizosphere soil. The PCoA, which was performed to determine the variability in the fungal community results between irrigation practices, showed that axes I and II respectively explained 53.6% and 23.9% of the variability (i.e., 77.5% of the variability in the fungal community distribution characteristics was explained by the first two axes) (Fig 3). Additionally, the PERMANOVA (distance-based) test revealed significant differences among DIR, DIB, CKR, and CKB (R^2^ = 0.92, P = 0.001). This finding reflects the significant differences in the fungal community structures between irrigation practices as well as the significant differences between rhizosphere and non-rhizosphere fungal communities under the same irrigation conditions.

**Fig 4:**
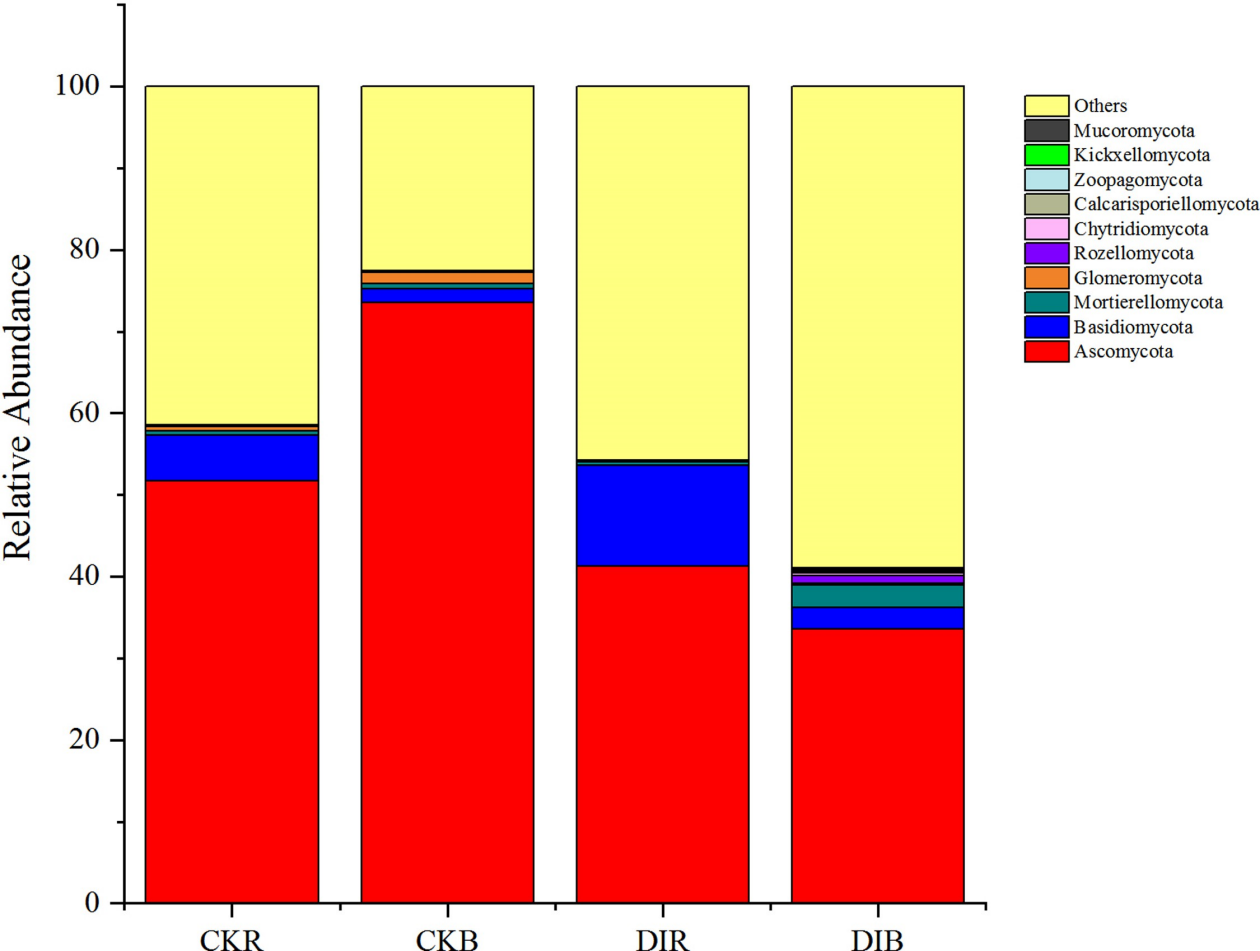
Distribution of fungal communities in response to different irrigation methods (phylum level).

The 10 most abundant fungal phyla were Ascomycota (50.04%), Basidiomycota (5.47%), Mortierellomycota (1.07%), Glomeromycota (0.55%), Rozellomycota (0.27%), Chytridiomycota (0.14%), Zoopagomycota (0.08%), Calcarisporiellomycota (0.05%), Kickxellomycota (0.03%), and Mucoromycota (0.03%) (Fig 4). Thus, there were six dominant phyla (>0.1%).

In rhizosphere soils, the relative abundance of Basidiomycota was significantly higher after drip irrigation than after flood irrigation, whereas the relative abundances of Ascomycota and Glomeromycota were significantly higher after flood irrigation than after drip irrigation (S1 Fig). In drip-irrigated soils, the relative abundances of Ascomycota and Basidiomycota were significantly higher in rhizosphere soil than in non-rhizosphere soil. In contrast, the relative abundances of Mortierellomycota and Rozellomycota were significantly higher in non-rhizosphere soil than in rhizosphere soil (S1 Fig).

**Fig 5:**
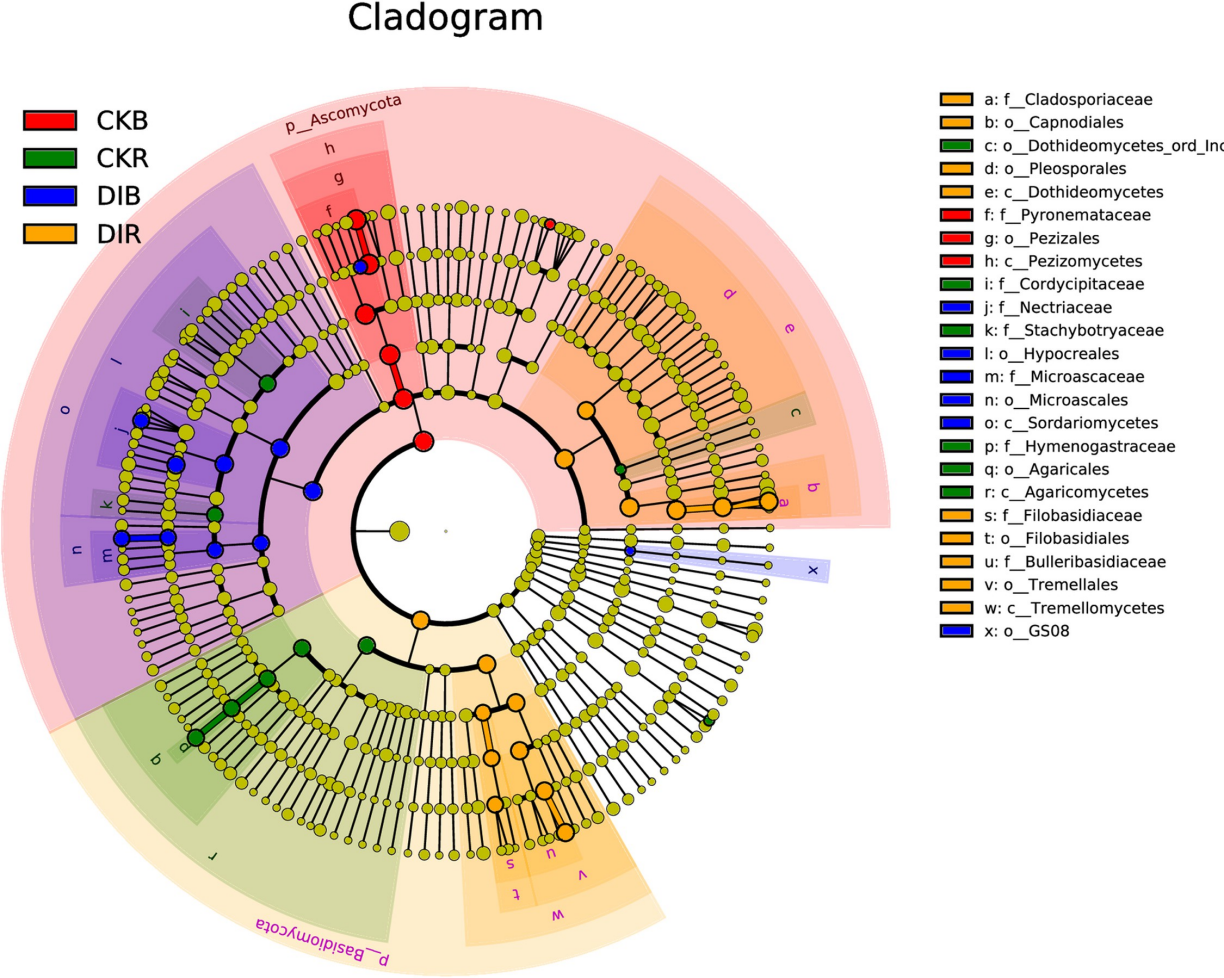
Cladogram of soil fungal populations in different soil samples. The linear discriminant analysis score was 4.0.

Significantly different biomarkers between soil samples were detected on the basis of LEfSe (Fig 5). In DIR, the iconic fungal populations were *Cladosporiaceae* (20.8%), *Bulleribasidiaceae* (8.73%), and *Filobasidiaceae* (2.37%). In DIB, the iconic fungal populations were *Nectriaceae* (13.09%) and *Microascaceae* (2.35%). In CKR, the iconic fungal populations were *Hymenogastraceae* (3.51%), *Cordycipitaceae* (3.45%), and *Stachybotryaceae* (2.17%). In CKB, the iconic fungal populations were *Rhodothermaceae* (3.95%) and *Pyronemataceae* (61.19%).

### Functional differences in soil fungal communities between irrigation practices

We annotated fungi using the FUNGuild database and identified 13 fungal functional groups, including Ectomycorrhizal (22.29%), Endophyte (9.40%), Plant_Pathogen (5.73%), Undefined_Saprotroph (7.83%), Animal_Pathogen (4.11%), Fungal _Parasite (2.81%), and Arbuscular_Mycorrhizal (0.55%).

**Fig 6:**
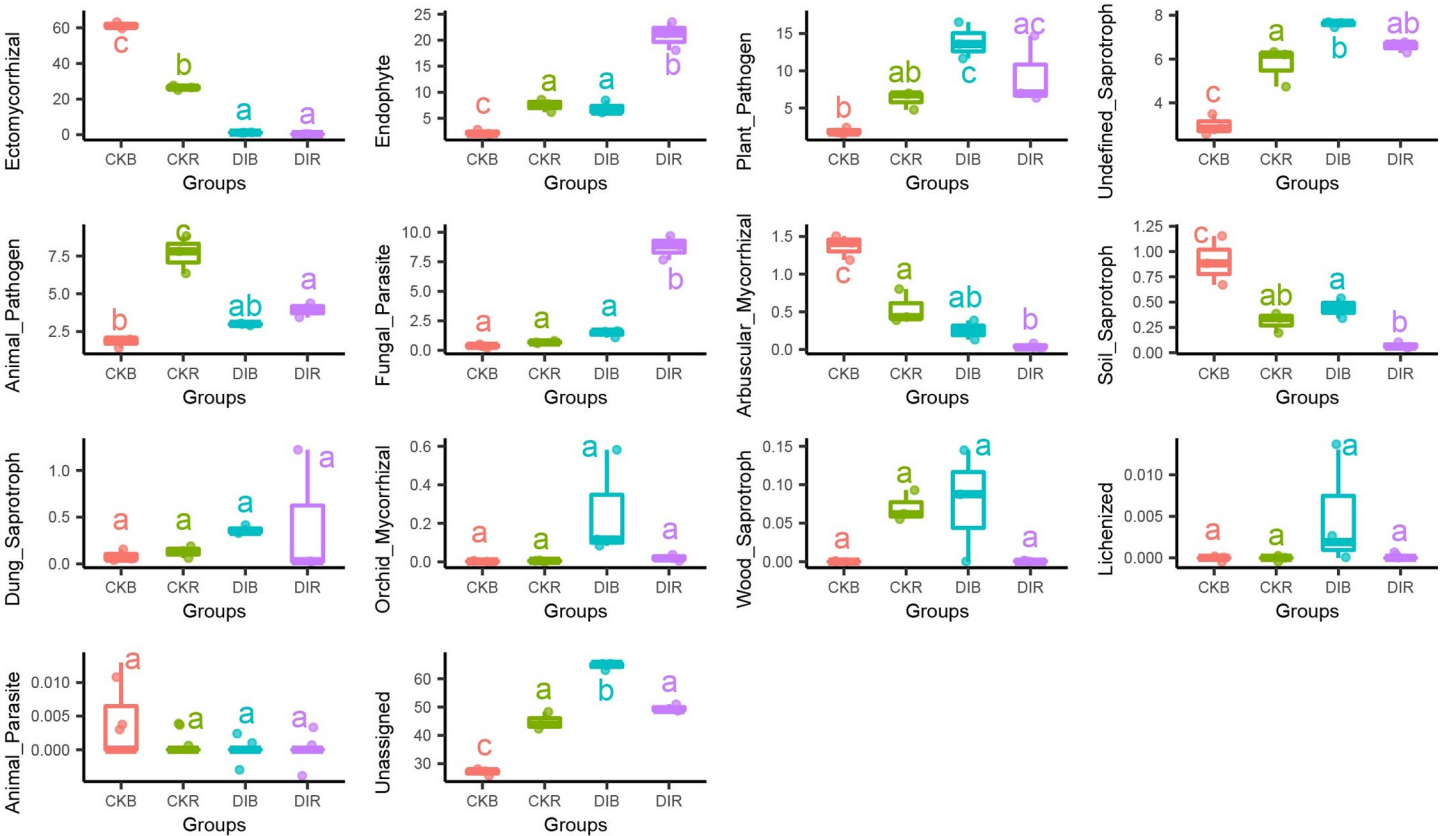
Functional analysis of soil fungi using FUNGuild. Data on the y-axis represent the relative abundance (%).

In rhizosphere soils, Endophyte and Fungal_Parasite were significantly more abundant after drip irrigation than after flood irrigation. The relative abundances of Ectomycorrhizal, Arbuscular_Mycorrhizal, and Animal_Pathogen were significantly higher after flood irrigation than after drip irrigation. The relative abundance of Plant_Pathogen was higher after drip irrigation than after flood irrigation, but not significantly (P = 0.19) (Fig 6). In drip-irrigated soils, Endophyte and Fungal_Parasite were significantly more abundant in rhizosphere soils than in non-rhizosphere soils, whereas Soil_Saprotroph was significantly more abundant in non-rhizosphere soils than in rhizosphere soils (Fig 6).

### Relationship between soil properties and fungal community structures

**Fig 7:**
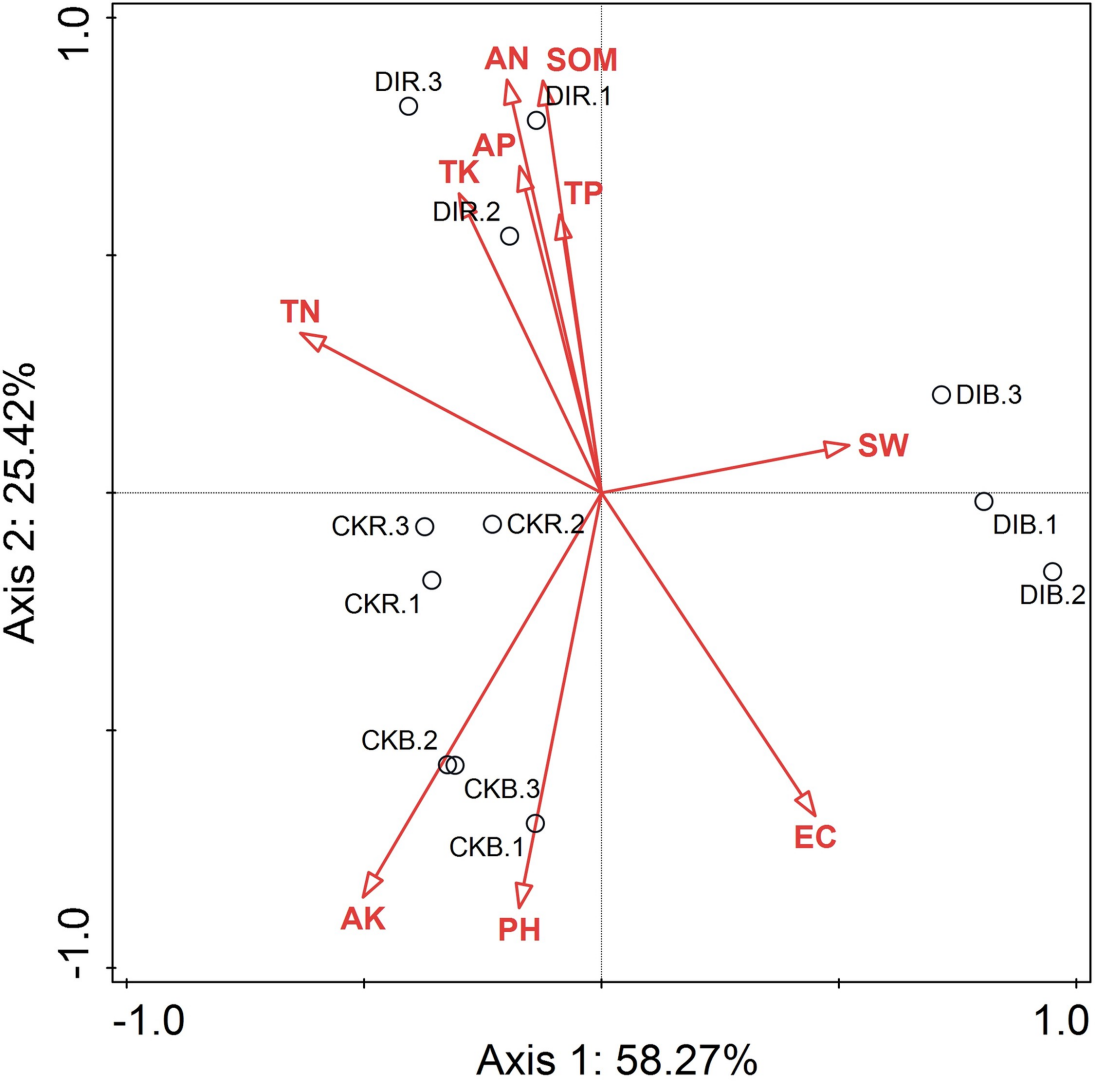
Redundancy analysis of soil fungal communities and environmental factors.

A redundancy analysis of soil physicochemical factors and fungal community structures was performed (Fig 7). The effect of soil physicochemical factors on the distribution of soil fungal communities is typically represented by arrows in the redundancy analysis, with the length of the arrows proportional to the degree of the correlation. The first and second axes respectively explained 58.27% and 25.42% of the variance in the fungal community structures (i.e., 83.69% in total). Thus, the first two axes reflected the overall soil fungal community distribution characteristics and the influencing factors. On the basis of the arrow lengths, AK had the greatest influence on the soil fungal community distribution characteristics, followed by TN (Fig 7).

The Monte Carlo test was conducted to verify the accuracy of the redundancy analysis. The rank order for the importance of soil environmental factors was as follows (Table 1): AK > AN > EC > SOM > TP > TK > pH > AP > SW > TN. The effects of AK, AN, and EC (33.1%, 38.2%, and 8.6%, respectively) were significant (P < 0.05), implying that AK, AN, and EC were the main factors affecting the soil fungal community structure.

**Table 1:** Monte Carlo test of the environmental variables and fungi.

Name	Rank	Explains %	pseudo-F	P
AK	1	33.1	5	0.008
AN	2	38.2	12	0.002
EC	4	8.6	4.7	0.004
SOM	3	7.2	2.7	0.066
TP	5	2.3	1.3	0.324
TK	6	1.9	1.1	0.412
PH	7	1.7	0.9	0.474
AP	8	1.5	0.8	0.517
SW	9	0.7	0.3	0.828
TN	10	2	0.7	0.596

**Fig 8:**
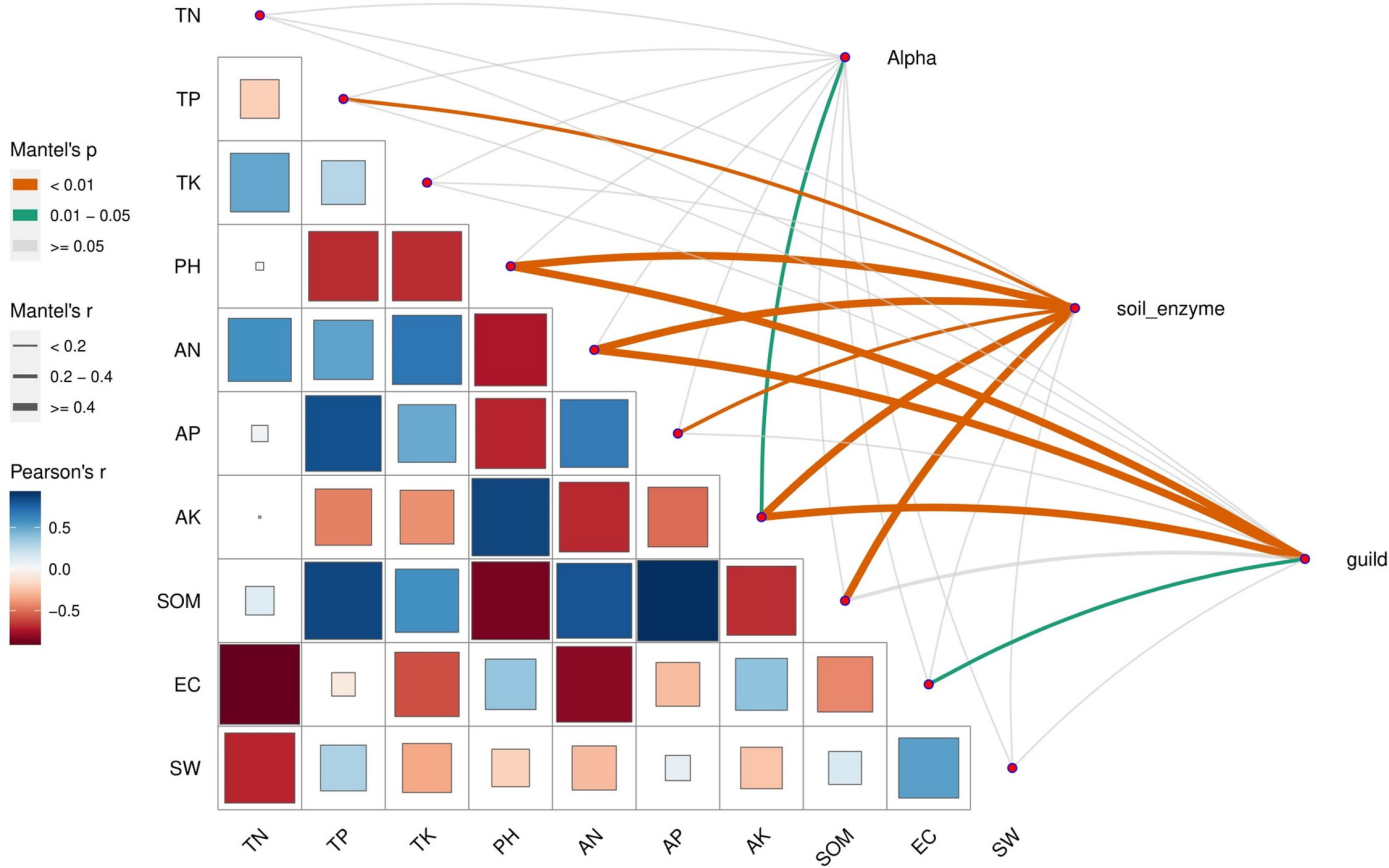
Correlation between fungal community structures (phylum level) and soil physicochemical properties. The lower left corner presents the correlations (Pearson) among the 10 soil environmental factors. The top right corner presents the correlations (Mantel) between soil environmental factors and soil enzyme activities, fungal alpha diversity, and fungal functions. Alpha: soil fungal alpha diversity; soil_enzyme: soil enzyme activities; guild: soil fungal functions.

Similarly, we identified the soil environmental factors affecting soil enzyme activities, fungal alpha diversity, and fungal functions under different irrigation conditions (Fig 8). There were strong correlations among environmental factors, with pH and AN having the greatest influence on the other environmental factors. The Mantel test results indicated only AK significantly affected the fungal alpha diversity, whereas TP, pH, AN, AP, AK, and SOM were significantly correlated with soil enzyme activities. Additionally, pH, AN, AK, and EC were significantly correlated with soil fungal functions. Overall, AK, AN, and pH were most closely associated with soil enzyme activities, fungal alpha diversity, and fungal functions.

## Discussion

### Effect of drip irrigation on soil fungal diversity

In soil ecosystems, soil fungal communities play a key role in soil nutrient cycling and plant growth and development [23, 24]. A previous study revealed significant differences in the microbial communities of flood-irrigated and drip-irrigated soils [25]. Similarly, in this study, the soil fungal alpha diversity, community structures, and functions differed significantly between drip irrigation and flood irrigation. There were also significant differences in the soil fungal communities between the drip-irrigated rhizosphere and non-rhizosphere soils.

Earlier research demonstrated that soil microbe diversity and the associated soil enzyme activities are influenced by irrigation practices, including drip irrigation [26]. Moreover, a highly diverse soil fungal community is indicative of a stable soil ecosystem and increased plant resistance to pathogens [27]. In this study, the alpha diversity indices (Shannon, OTUs, and Chao1) were significantly lower for the drip-irrigated rhizosphere soil than for the flood-irrigated rhizosphere soil. Hence, drip-irrigated alfalfa may be less resistant to pathogens than flood-irrigated alfalfa. The SUE and SNP activities can reflect N and P decomposition and conversion in the soil [28]. In the present study, compared with flood irrigation, drip irrigation significantly increased the SUE and SNP activities, which may indicate that drip irrigation significantly increased the effectiveness of N and P in the alfalfa rhizosphere soil. An earlier investigation confirmed that drip irrigation affects soil environmental factors and significantly influences the soil microorganism diversity [29]. Similarly, in this study, AK significantly affected soil fungal alpha diversity. Irrigation results in alternating wet and dry soils, leading to changes in root secretions [30]. Complex root-secreted compounds have critical effects on rhizosphere soil fungal communities [31–33], which may explain the marked differences in the rhizosphere and non-rhizosphere soil fungal diversity observed in the present study. Thus, compared with flood irrigation, drip irrigation significantly increases the activity of urease and neutral phosphatase in the rhizosphere soil, but it significantly decreases the alpha diversity of rhizosphere soil fungi.

### Effect of drip irrigation on soil fungal community structures

Ascomycota was identified as the dominant phylum. The fungi belonging to this taxonomic group are usually highly adaptable, widely distributed, and the main fungal species in various soils. The findings of this study are consistent with those of earlier studies on the soil fungal community structure in arid regions [34, 35]. According to Wang and Huang, different irrigation methods significantly affect soil fungal communities in paddy fields [36]. In accordance with this earlier finding, we detected differences in the fungal communities of drip-irrigated and flood-irrigated soils. Furthermore, the relative abundances of Ascomycota and Glomeromycota were significantly higher in CKR than in DIR. Ascomycota fungal species are mostly saprophytic and have important roles related to the degradation of SOM [37]. Many Glomeromycota species can form a symbiotic relationship with plants, thereby promoting nutrient uptake and significantly enhancing plant growth and development [38]. Compared with flood-irrigated soil, nutrient cycling may be slower and mycorrhizal symbioses involving alfalfa plants may be more limited in drip-irrigated soils.

On the basis of the LEfSe analysis, the iconic fungi in DIR mainly belonged to the families Cladosporiaceae, Bulleribasidiaceae, and Filobasidiaceae, with Cladosporiaceae detected as the iconic fungal group with the highest relative abundance at the family level in DIR. This family consists of a complex group of saprophytic fungi that exist in diverse environments. Additionally, many of the fungi in this family are phytopathogens. The iconic fungi in CKR belonged to Hymenogastraceae, Cordycipitaceae, and Stachybotryaceae, of which Cordycipitaceae consists of entomopathogenic fungi that can release biotoxins as well as infest insects. Thus, it may be useful for pest control [39]. Furthermore, many Glomeromycetes species are beneficial fungi that can form mycorrhizal structures in plants [38, 40, 41]. In this study, the relative abundance of Glomeromycetes was significantly lower in DIR than in CKR, indicating that drip irrigation may inhibit the formation of mycorrhizal symbionts. Overall, compared with the effects of flood irrigation, drip irrigation significantly modified the composition of the soil fungal community and decreased the abundance and diversity of soil fungi.

### Effect of drip irrigation on soil fungal functions

FUNGuild is an emerging platform useful for the functional annotation of fungi in specific ecosystems, including agricultural soils [42]. Fungal functional groups have important effects on the geochemical cycles and energy fluxes of ecosystems, and they are closely linked to soil nutrient balance and plant development [43]. During the functional annotation in the current study, ectomycorrhizal fungi were identified as the primary species. Previous studies determined that ectomycorrhizal fungi may play an active role in organic matter decomposition, with large networks of ectomycorrhizal fungi positively affecting soil C and N cycling [44, 45], implying soil C and N contents are crucial factors modulating alfalfa growth. Additionally, Arbuscular_Mycorrhizal was significantly less abundant in drip-irrigated soil than in flood-irrigated soil, which is similar to the differences in the abundance of Glomeromycota following drip irrigation and flood irrigation [38]. Similar to previous studies, Klimek et al. analyzed the effects of three irrigation methods and observed that subsurface drip-irrigated soils have the lowest mycorrhizal content [46]. This may be because compared with flood irrigation, drip irrigation significantly decreases the loss of soil nutrients [47], resulting in a significant increase in the soil N and P concentrations. This increase in the soil N and P contents may significantly inhibit the growth of mycorrhizal fungi [48, 49]. In this study, there was no significant difference in Plant_Pathogen between the drip-irrigated soil and the flood-irrigated soil, which is in accordance with the findings of an earlier study [50]. However, although the difference was not significant (P = 0.19), the slightly higher relative abundance of Plant_Pathogen in drip-irrigated soil than in flood-irrigated soil may lead to an increased risk of disease in drip-irrigated alfalfa. Therefore, compared with flood irrigation, drip irrigation significantly decreases the abundance of Ectomycorrhizal and Arbuscular_Mycorrhizal, but it may have the opposite effect on the abundance of Plant_Pathogen.

### Soil environmental factors and their effects on the structural composition of fungal communities

Irrigation is one of the most important sources of soil moisture in arid zones [51]. Previous studies indicated that different irrigation methods have important effects on plants and the availability of nutrients in the soil surrounding plant roots [52]. Li et al. detected significantly higher nutrient levels in drip-irrigated soils than in conventional flood-irrigated soils in an arid zone [53]. Similarly, in the present study, the TP, TK, SOM, AN, and AP contents were significantly higher in drip-irrigated soil than in flood-irrigated soil, which may indicate that drip irrigation is better than flood irrigation for decreasing soil nutrient leaching [47].

Previous studies have shown that soil physical and chemical properties are important factors influencing the distribution of soil fungal communities and that changes in soil environmental factors can alter soil fungal communities and soil enzyme activities [54, 55]. In this study, the redundancy analysis of soil environmental factors and fungal community structures revealed that AK, AN, and EC are three key physicochemical factors modulating the composition of fungal communities. Similarly, among the examined factors, AK, AN, and pH were found to be most strongly associated with soil enzyme activities, fungal alpha diversity, and fungal functions according to the Mantel test. Both AK and AN (i.e., soil N and K readily available for plant uptake) are important for plant growth and development. Because of co-evolution, AK and AN may also be important factors influencing the soil fungal community. Additionally, earlier research confirmed that soil pH is a critical factor regulating the composition and diversity of soil fungal communities [56]. The composition and distribution of fungal communities in soils are sensitive to soil pH. It is believed that pH can influence the transformation and synthesis of other soil factors, enabling it to indirectly modify the fungal community structure [57]. Moreover, the Xinjiang region mainly consists of saline-alkaline soil. Accordingly, drip irrigation may be useful for gradually decreasing the soil pH and for inducing the accumulation of nutrients in the soil, both of which will lead to increased soil quality. Overall, AK and AN are the main environmental factors affecting soil enzyme activities and fungal community structures in response to different irrigation practices.

## Conclusion

In the arid and saline Tianshan agricultural region, where flood irrigation is the traditional irrigation method, the use of drip irrigation has been promoted to improve water use efficiency. However, relatively little is known about the effects of drip irrigation on the rhizosphere soil enzyme activities and fungal communities of economically important crops in this region. In this study, alfalfa soil physicochemical properties, enzyme activities, and fungal communities differed significantly between the drip and flood irrigation treatments. Specifically, AN and AP contents as well as enzyme activities were significantly higher in the drip-irrigated alfalfa rhizosphere soil than in the flood-irrigated alfalfa rhizosphere soil, suggesting drip irrigation may be conducive to alfalfa growth. However, compared with flood irrigation, drip irrigation significantly decreased the abundance of beneficial functional fungi, including ectomycorrhizal and arbuscular mycorrhizal species, and increased the abundance of pathogenic fungi. Therefore, drip irrigation may increase the risk of disease in alfalfa roots. Additionally, AK was revealed to be an important soil environmental factor affecting soil enzyme activities as well as fungal alpha diversity, community structures, and functions. Overall, compared with flood irrigation, drip irrigation significantly increases soil enzyme activities, but it significantly decreases the abundance of beneficial fungal communities, while also increasing the abundance of pathogenic fungal communities, which may increase the risk of alfalfa infections.

## Supporting information

S1 Fig Comparison of dominant fungal phyla. (TIFF)
