# Correction: Influence of different irrigation methods on the alfalfa rhizosphere soil fungal communities in an arid region

**DOI:** 10.1371/journal.pone.0307318

**Published:** 2024-07-11

**Authors:** Qizhang Deng, Yong Wu, Xiang Zhao, Chengshu Qiu, Shan Xia, Yuanyuan Feng, Hongling Liu

There are errors in the author affiliations. The correct affiliations are as follows:

Qizhang Deng^1,2^, Yong Wu^1^, Xiang Zhao^2^, Chengshu Qiu^1^, Shan Xia^1^, Yuanyuan Feng^1^, Hongling Liu^1^

**1** Sichuan Provincial Key Laboratory for Development and Utilization of Characteristic Horticultural Biological Resources, College of Chemistry and Life Sciences, Chengdu Normal University, Chengdu, Sichuan Province, 611130, China, **2** College of Life Sciences, Shihezi University, Shihezi, Xinjiang Province, 832003, China.
