# Supplementary figures and images for: Influence of different irrigation methods on the alfalfa rhizosphere soil fungal communities in an arid region
Source: PLoS One. 2022 Jun 17;17(6):e0268175. doi: 10.1371/journal.pone.0268175 (PMC9205495; doi:10.1371/journal.pone.0268175)

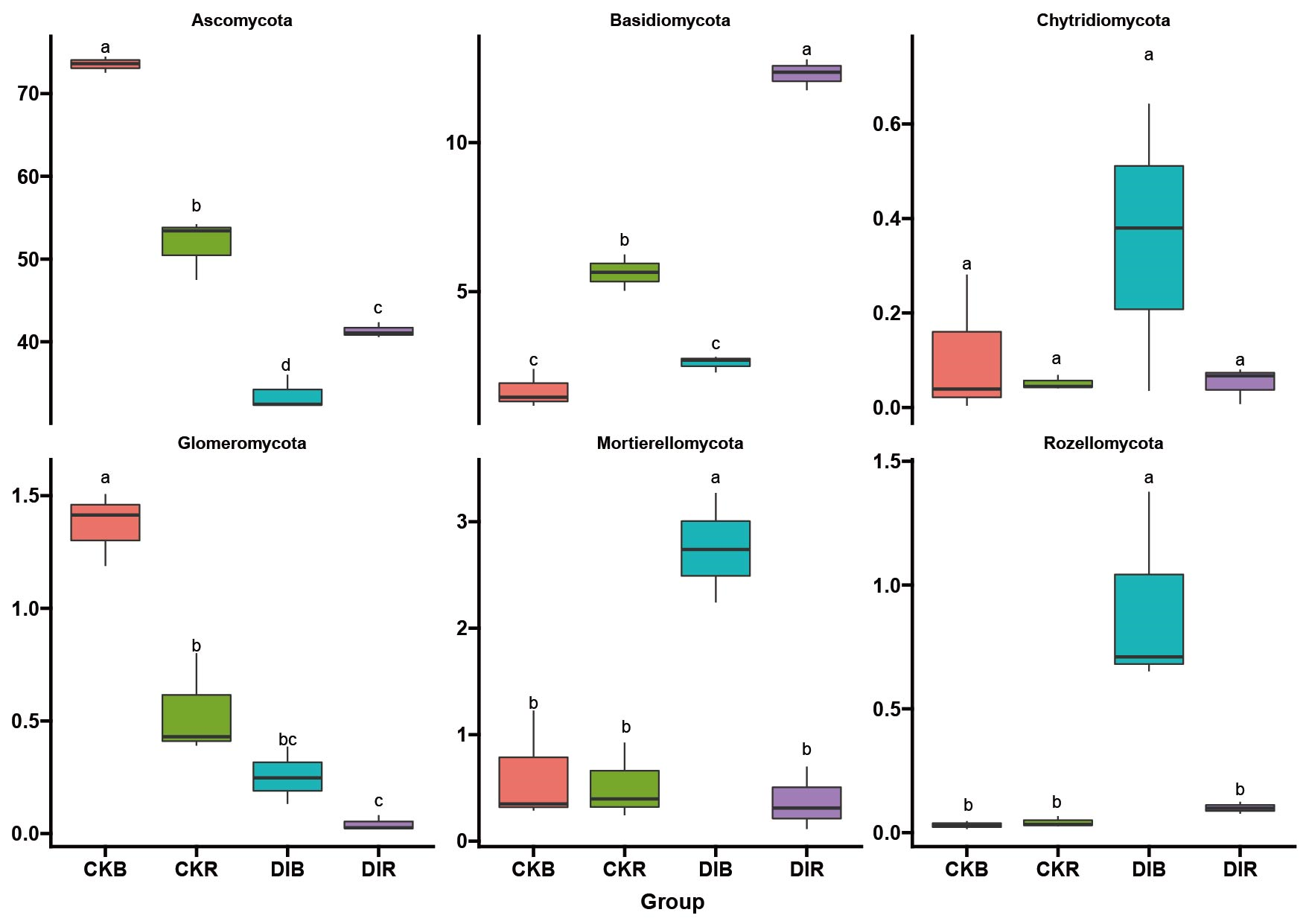

Supplement: S1 Fig — (TIFF) [file pone.0268175.s001.tiff]
